# Serum metabolomics in chickens infected with *Cryptosporidium baileyi*

**DOI:** 10.1186/s13071-021-04834-y

**Published:** 2021-06-26

**Authors:** Xue-Mei Wu, Xin Yang, Xian-Cheng Fan, Xi Chen, Yu-Xin Wang, Long-Xian Zhang, Jun-Ke Song, Guang-Hui Zhao

**Affiliations:** 1 Department of Parasitology, College of Veterinary Medicine, Northwest A&F University, Yangling, 712100 People’s Republic of China; 2 Center of Animal Disease Prevention and Control of Huyi District, Xi’an, 710300 People’s Republic of China; 3 College of Veterinary Medicine, Henan Agricultural University, Zhengzhou, 450046 People’s Republic of China

**Keywords:** Chicken, *Cryptosporidium baileyi*, Serum sample, Metabolomics, Pathway analysis

## Abstract

**Background:**

*Cryptosporidium baileyi* is an economically important zoonotic pathogen that causes serious respiratory symptoms in chickens for which no effective control measures are currently available. An accumulating body of evidence indicates the potential and usefulness of metabolomics to further our understanding of the interaction between pathogens and hosts, and to search for new diagnostic or pharmacological biomarkers of complex microorganisms. The aim of this study was to identify the impact of *C. baileyi* infection on the serum metabolism of chickens and to assess several metabolites as potential diagnostic biomarkers for *C. baileyi* infection.

**Methods:**

Ultraperformance liquid chromatography-mass spectrometry (UPLC-MS) and subsequent multivariate statistical analysis were applied to investigate metabolomics profiles in the serum samples of chickens infected with *C. baileyi*, and to identify potential metabolites that can be used to distinguish chickens infected with *C. baileyi* from non-infected birds.

**Results:**

Multivariate statistical analysis identified 138 differential serum metabolites between mock- and *C. baileyi*-infected chickens at 5 days post-infection (dpi), including 115 upregulated and 23 downregulated compounds. These metabolites were significantly enriched into six pathways, of which two pathways associated with energy and lipid metabolism, namely glycerophospholipid metabolism and sphingolipid metabolism, respectively, were the most enriched. Interestingly, some important immune-related pathways were also significantly enriched, including the intestinal immune network for IgA production, autophagy and cellular senescence. Nine potential *C. baileyi*-responsive metabolites were identified, including choline, sirolimus, all-*trans* retinoic acid, PC(14:0/22:1(13Z)), PC(15:0/22:6(4Z,7Z,10Z,13Z,16Z,19Z)), PE(16:1(9Z)/24:1(15Z)), phosphocholine, SM(d18:0/16:1(9Z)(OH)) and sphinganine.

**Conclusions:**

This is the first report on serum metabolic profiling of chickens with early-stage *C. baileyi* infection. The results provide novel insights into the pathophysiological mechanisms of *C. baileyi* in chickens.

**Graphic abstract:**

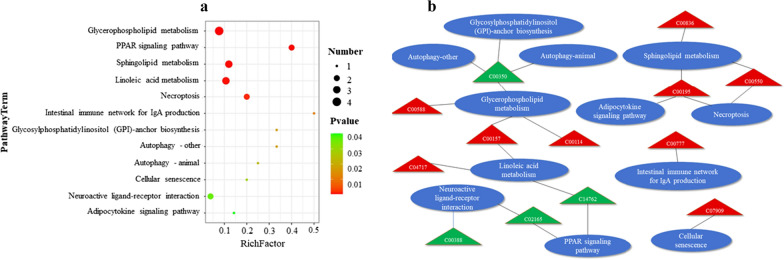

**Supplementary Information:**

The online version contains supplementary material available at 10.1186/s13071-021-04834-y.

## Background

*Cryptosporidium*, an important zoonotic protozoan parasite of humans and animals, has been reported in approximately 30 avian species worldwide, with the prevalence ranging from 0.8 to 44.4% [1]. Among the *Cryptosporidium* species reported in birds, *C. baileyi* is the dominant species across all continents, with the exception of Antarctica, especially in chickens [2, 3]. *C. baileyi* infection can cause serious respiratory symptoms (e.g. coughing, sneezing and dyspnea), decrease in weight gain, higher morbidity and mortality in chickens [4, 5]. Notably, co-infections of *C. baileyi* with other respiratory and gastrointestinal pathogens (e.g. *Escherichia coli* and infectious bronchitis virus) have also been reported in chickens [6, 7]. Further, an immunosuppressive effect of *C. baileyi* infection was also shown following vaccination against reoviruses, infectious bronchitis virus, Newcastle disease virus and avian influenza virus, resulting in significant economic losses to the poultry industry [7–9]. More significantly, *C. baileyi* has been detected in immunodeficient patients [10, 11], suggesting the potential zoonotic risk of this species. However, no effective preventive or treatment agents against *C. baileyi* infection have been developed to date [12].

The identification of *C. baileyi* infection is currently mainly based on the presence of oocysts in host feces or of other developmental stages (e.g. schizonts/merozoites, gametocytes/gametes) during histopathological observation [13, 14]. However, the performance of oocyst-based procedures to determine *C. baileyi* infection is often delayed due to their destructive effects on parasitized tissues (e.g. trachea, bursa of Fabricius, and cloaca), thus relying on the numbers and depth of schizonts prior to oocyst formation [4, 13]. Invasive or injuring histopathological observation is an unattractive option for antemortem diagnosis [14]. In recent decades, metabolomics, an increasingly recognized approach for identifying and quantifying multiple small chemical metabolites in complex biological samples, has been proved to be useful in the search for new diagnostic or pharmacological biomarkers of complex microorganisms (e.g. *Mycobacterium tuberculosis*, *Toxoplasma gondii*) [15, 16] as well as for improving our understanding of dormant and intricate interactions between hosts and pathogens [17–22]. Many compartments have been used to profile chemical metabolites in humans and animals, such as feces, saliva, urine and blood [23]. Although urine would be the better choice of biofluid in terms of developing a non-invasive diagnostic marker [24], serum samples are more suitable for tracking metabolites with the aim to develop non-invasive diagnostic markers for birds (e.g. chicken) since it’s difficult to purify urine from fecal mixtures due to the specific structural characteristics of the digestive system of birds [25, 26]. Numerous analytical platforms are also applied in metabolomic studies, including nuclear magnetic resonance (NMR) and gas or liquid chromatography coupled to mass spectrometry (GC–MS/LC–MS) [27]. Compared with GC–MS and NMR, LC–MS currently represents the major instrumental and analytical technology used in the global profiling of metabolites [28], and it enables rapid quantification of multiple metabolites ranging from nanograms per liter to grams per liter in body fluids [29]. To further improve the resolution and sensitivity of LC–MS, ultraperformance liquid chromatography-MS (UPLC-MS), one of the more versatile techniques, has been established and used in metabolomics studies [30–32]. Previous studies have reported metabolic alterations associated with intestinal permeability in mouse feces following infection with *C. parvum* using GC–MS [33] and diarrheal aspects of the disease in the mouse gut and COLO-680 N cells during infections of *C. parvum* or *C. hominis* [34]. In the present study, we explored the serum metabolomic profiles of chickens in the early stages of *C. baileyi* infection.

## Methods

### Chemicals and reagents

All chemicals and solvents were analytical grade. Methanol, acetonitrile and formic acid were purchased from CNW Technologies GmbH (Düsseldorf, Germany). 2-Chloro-L-phenylalanine was obtained from Shanghai Hengchuang Bio-technology Co., Ltd. (Shanghai, China). 1-Heptadecanoyl-2-hydroxy-*sn*-glycero-3-phosphocholine [LysoPC(17:0)] was purchased from Avanti Polar Lipids (Alabaster, AL, USA).

### Experimental design

A total of 18 newly hatched white cockerels (Hy-line variety) were purchased from the Giant Long Company (Shaanxi, China) and randomly divided into experimental (Case 1, E1–E9: *C. baileyi*-infected chickens) and mock (Con 1, N1–N9) groups with nine chickens per group. Animal care was according to the recommendations of the National Research Council (NRC) as published in the Guide for the Care and Use of Laboratory Animals [35]. All chickens had free access to clean feed and sterile water throughout the whole experimental period. The base diet was also per the recommendation of the NRC, and the components and nutrient levels of the diets are listed in Additional file 1: Table S1. Chickens in the experimental group were orally infected with 1 × 10^6^ *C. baileyi* oocysts at 3 days after birth according to our previous study [36], while mock birds were orally inoculated with the same volume of phosphate buffer saline.

### Sample collection

All chickens were in good nutritional status during the entire experimental period, and no birds died. The blood sample was collected from the heart of each chicken simultaneously into a separate Eppendorf tube to isolate the serum sample on the afternoon of day 5 post-infection (dpi). These samples were used for analyzing the profiles of early serum metabolites since *C. baileyi* oocyst shedding in chicken feces began at 5 dpi. The obtained serum sample was transferred into a new Eppendorf tube and stored immediately at − 80 °C for further analysis. All chickens in both groups were sacrificed by cervical dislocation, sterilized and stored in specific bags for biological wastes and then later recovered for further processing by the Laboratory Animal Center of Northwest A&F University.

### Confirmation of infection

Fecal samples of all chickens in both groups were examined daily using Sheather's sucrose flotation technique [37] and *18S* rRNA gene-based nested PCR as reported in [38] to confirm *C. baileyi* infection. Oocysts per gram feces (OPGs) were counted using the hemocytometer.

### Sample preparation for UPLC-MS analysis

For each blood collection, a 100-μl sample of serum was mixed with 10 μl of 2-chloro-L-phenylalanine (0.3 mg/mL) (internal standard) and vortexed for 10 s, followed by the addition of 300 μl of an ice-cold mixture of methanol and acetonitrile (2:1, v/v). The mixed solution was vortexed for 1 min, ultrasonicated in an ice-water bath for 10 min, kept at − 20 °C for 30 min and then centrifuged at 13,000 rpm for 10 min at 4 °C. The supernatant from each tube was then collected and filtered through a 22-μm organic phase pinhole microfilter. Finally, the filtrate was transferred into a UPLC vial and stored at − 80 °C for subsequent UPLC-MS analysis. All extraction reagents were precooled at − 20 °C in advance. Quality control (QC) samples were pooled by mixing all samples at an equal volume.

### UPLC–MS analysis for untargeted metabolite profiling

The UPLC-MS analysis was performed on an ACQUITY UPLC I-Class system (Waters Corp., Milford, MA, USA) coupled with a Xevo G2-XS QTof mass spectrometer (Waters Corp.). A 2-μl aliquot of the filtrate described in the previous section was injected into an ACQUITY UPLC BEH C18 column (1.7 μm, 2.1 mm × 100 mm; Waters Corp.) at a flow rate of 0.4 ml/min and a column oven of 45 °C. Mobile phases A and B were water/formic acid (99.9:0.1, v/v) and acetonitrile/formic acid (99.9:0.1, v/v), respectively. Metabolite elution was conducted at the following linear gradient: 0 min, 1% (B); 1 min, 5% (B); 2 min, 30% (B); 3.5 min, 60% (B); 7.5 min, 90% (B); 9.5–12.5 min, 100% (B); 12.7–16 min, 1% (B). All samples were kept at 4 °C during the analysis.

The MS data were acquired in a centroid mean square error (MSE) mode with an electrospray ionization (ESI) source operating in either positive or negative ion mode. The capillary voltage was set to 3 and 2 kV for positive and negative ions, respectively; the sampling cone voltage was set to 40 V. The time-of-flight (TOF) mass range was set from 50 to 1000 *m/z*, and the scan time was 0.1 s. The QCs were injected at regular intervals (every 9 samples) throughout the analytical run to assess repeatability of the data. All procedures were carried out in the laboratory of Shanghai Luming Biotechnology Co., Ltd. (Shanghai, China).

### Data processing and statistical analysis

Raw data were collected from the UPLC-MS analysis platform and preprocessed using the software progenesis QI v2.3 (Nonlinear Dynamics, Newcastle, UK), including baseline filtering, peak picking, integration, retention time (RT) alignment, peak alignment and normalization. The main parameters were set as follows: precursor tolerance, 5 ppm; product tolerance, 10 ppm; product ion threshold, 5%. The metabolites were qualitatively identified by alignment with the Human Metabolome Database (HMDB), Lipidmaps (v2.3) and METLIN Database. Three-dimensional data sets were integrated into an Excel file (Microsoft Corp., Redmond, WA, USA), including the mass-to-charge ratio (* m/z*), peak RT and peak intensities, and each ion was identified by using RT–*m/z* pairs. Any peak with missing values (ion intensity = 0) in > 50% of samples was removed to generate the final matrix. The internal standard was used for QC data (reproducibility). Data for both positive and negative ions were combined into a matrix table and imported into SIMCA software package (v14.0; Umetrics, Umeå, Sweden) for multivariate statistical analysis. Unsupervised principal component analysis (PCA) was utilized to visualize systemic variations and general clustering among all groups, and supervised partial least squares-discriminant analysis (PLS-DA) and orthogonal PLS-DA (OPLS-DA) were used to identify differential metabolites between the mock and experimental groups. The Variable Importance in Projection (VIP) scores in the OPLS-DA model were calculated to select potential differential metabolites, and the variables with a VIP value > 1 were considered relevant for group discrimination. Model overfitting was monitored by using the default seven-fold cross validation and 200-times response permutation testing (RPT). The differential metabolites between two groups were further screened by using the Benjamini–Hochberg adjusted Student’s *t*-test and fold-change analysis. The log_2_ fold change (log_2_FC) represented the ratio of the abundance of the average ion intensities in sera of infected chickens compared to that of mock birds. The *P* value adjusted by the Benjamini–Hochberg method, also known as the false discovery rate (FDR), was used to identify differentially expressed metabolites and metabolic pathways affected by *C. baileyi* infection [39]. Metabolites with a VIP value > 1 and FDR (*q*-value) < 0.05 were identified as significantly differential metabolites. Additionally, the Kyoto Encyclopedia of Genes and Genomes (KEGG; http://www.genome.jp/kegg/) was used to identify important pathways related to metabolic phenotypes following *C. baileyi* infection.

### Biomarker characterization and identification

To identify potential biomarkers for the early diagnosis of *C. baileyi* infection in chickens, we performed univariate receiver operating characteristic (ROC) curve analyses to quantify the predictive performance of the differential metabolites of interest, and the accuracy (including sensitivity and specificity) was evaluated for each selected metabolite using the area under the ROC curves (AUC), with AUC > 0.7 as the threshold.

## Results

### Confirmation of *C. baileyi* infection in chickens

Morphological observation of chicken feces under a microscope (ECLIPSE 80i; Nikon Corp., Tokyo, Japan) found *Cryptosporidium* oocysts in the feces of the experimental group by using Sheather's sucrose flotation technique without dye, but no oocysts were detected in the mock group (Additional file 2: Figure S1a). The *18S* rRNA gene of *Cryptosporidium* was amplified and sequenced in fecal samples of chickens in the experimental group (Additional file 2: Figure S1b), and the sequence identity was 100% to a *C. baileyi* isolate from a farmed chicken in Hubei Province, China (MG969393). Oocyst shedding in infected chickens was found from 5 to 28 dpi, with two OPG peaks at 10 and 16 dpi, respectively (Additional file 2: Figure S1c).

### Metabolite profiling of chicken serum samples

**Fig. 1:**
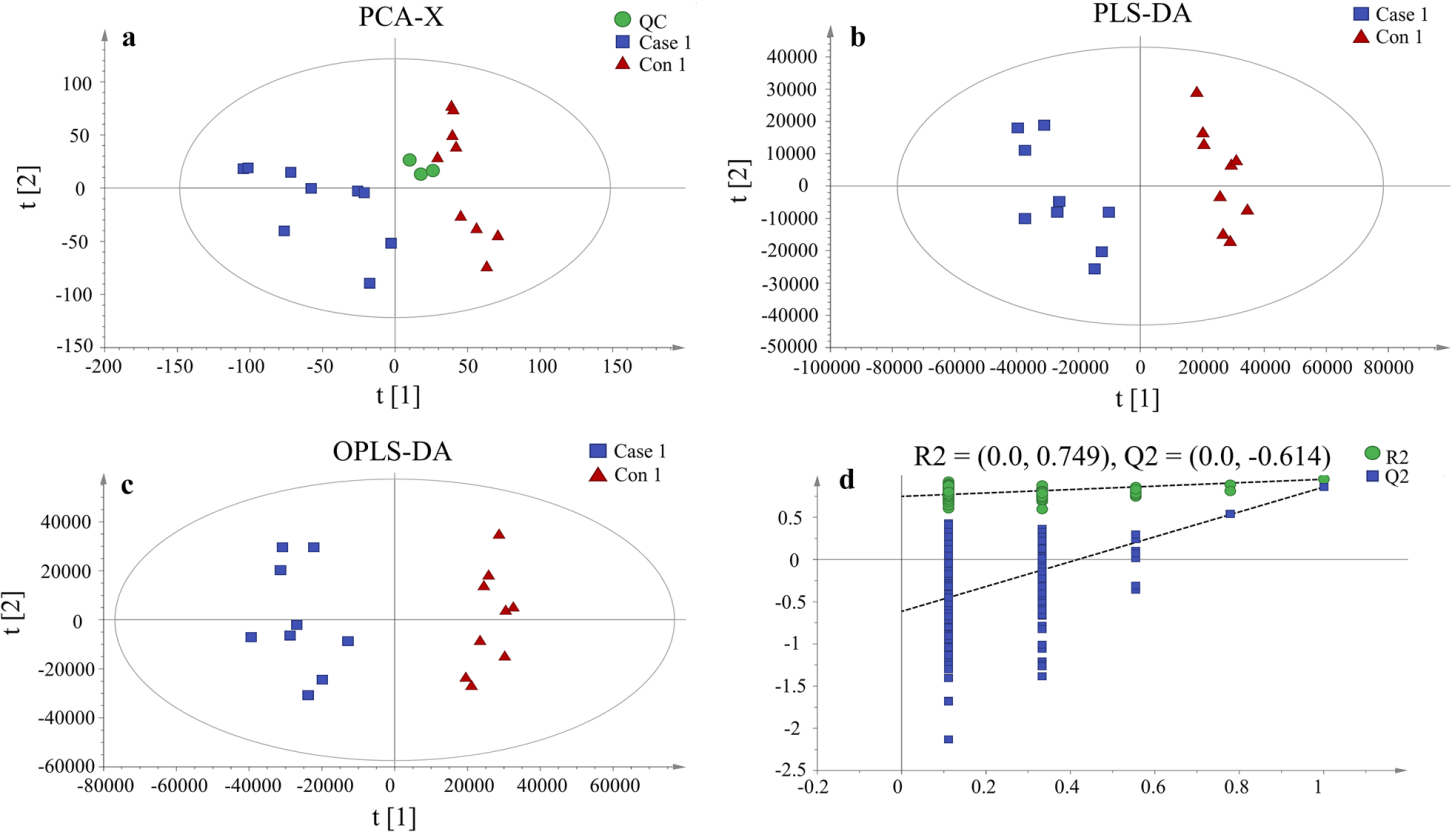
Score plots of multivariate statistical analysis. **a** Principal component analysis (PCA) score plots for all samples. Case 1 *Cryptosporidium baileyi*-infected chickens, Con 1 phosphate buffer saline-inoculated chickens, QC quality control. **b** Partial least squares-discriminant analysis (PLS-DA) score plots for Case 1 and Con 1 samples, **c** orthogonal partial least squares-discriminant analysis (OPLS-DA) score plots for Case 1 and Con 1 samples, **d** results of 200-times response permutation testing of OPLS-DA. Q2 and R2 represent the intercepts of the regression curve and y-axis generated by the linear regression between the R2 and Q2 values of "permuted” model and the R2Y and Q2Y values of the "real" OPLS-DA model, respectively

A total of 4235 metabolites were identified by UPLC-MS (1945 and 2290 for negative and positive ion modes, respectively) in serum samples from the mock and infected chicken groups at 5 dpi, of which 2807, 1147 and 281 were matched against HMDB, Lipidmaps (v2.3) and METLIN Database, respectively (Additional file 3: Table S2). Of these 4235 metabolites, 3865 were clustered into 24 classified super classes, including lipids and lipid-like molecules (2164), organoheterocyclic compounds (437), organic acids and derivatives (396), benzenoids (215), organic oxygen compounds (189), phenylpropanoids and polyketides (189), organic nitrogen compounds (58), alkaloids and derivatives (44), organosulfur compounds (40), organooxygen compounds (37), nucleosides, nucleotides and analogues (27), hydrocarbons (19), homogeneous non-metal compounds (14), organohalogen compounds (13), organometallic compounds (4), lignans, neolignans and related compounds (3), mixed metal/non-metal compounds (3), organic compounds (3), organonitrogen compounds (3), hydrocarbon derivatives (2), organic 1,3-dipolar compounds (2), inorganic compound (1), miscellaneous inorganic compound (1) and organophosphorus compound (1). The good stability and repeatability of the analysis were revealed by the sufficiently close positioning of the QC samples in a PCA score plot (Fig. 1a). Clear separations between the mock and *C. baileyi*-infected chicken sera were also shown on score plots of PCA, PLS-DA and OPLS-DA (Fig. 1b, c), indicating that the two experimental groups had distinct metabolic profiles. Seven-fold cross validation R2Y (0.95) and Q2 (0.86) showed good fitness and predictability, and the negative Q2 in the 200-times response permutation testing revealed no overfitting in OPLA-DA (Table 1; Fig. 1d).

**Table 1:** Each parameter of the multivariate statistical analysis

Samples	Models	R2X (cumulative)	R2Y (cumulative)	Q2(cumulative)	Q2
All	PCA-X	0.34		0.036	
Case 1–Con 1^a^	PLS-DA	0.37	0.95	0.81	
	OPLS-DA	0.37	0.95	0.86	− 0.61

R2Y metric describing the percentage of Y matric explained by the model, Q2 (cumulative) metric describing the predictive ability of the model, Q2 metric representing a parameter that describes whether the OPLS-DA opls-da model is over-fitted, *PCA* principal *component analysis*, *PLS-DA* partial least squares-discriminant analysis, *OPLS-DA* orthogonal partial least squares-discriminant analysis,

^a^Case 1: experimental group (*Cryptosporidium baileyi*-infected chickens); Con 1: mock-inoculated (with phosphate buffered saline) group

### Effect of *C. baileyi* infection on metabolite profiles in chicken sera

**Fig. 2:**
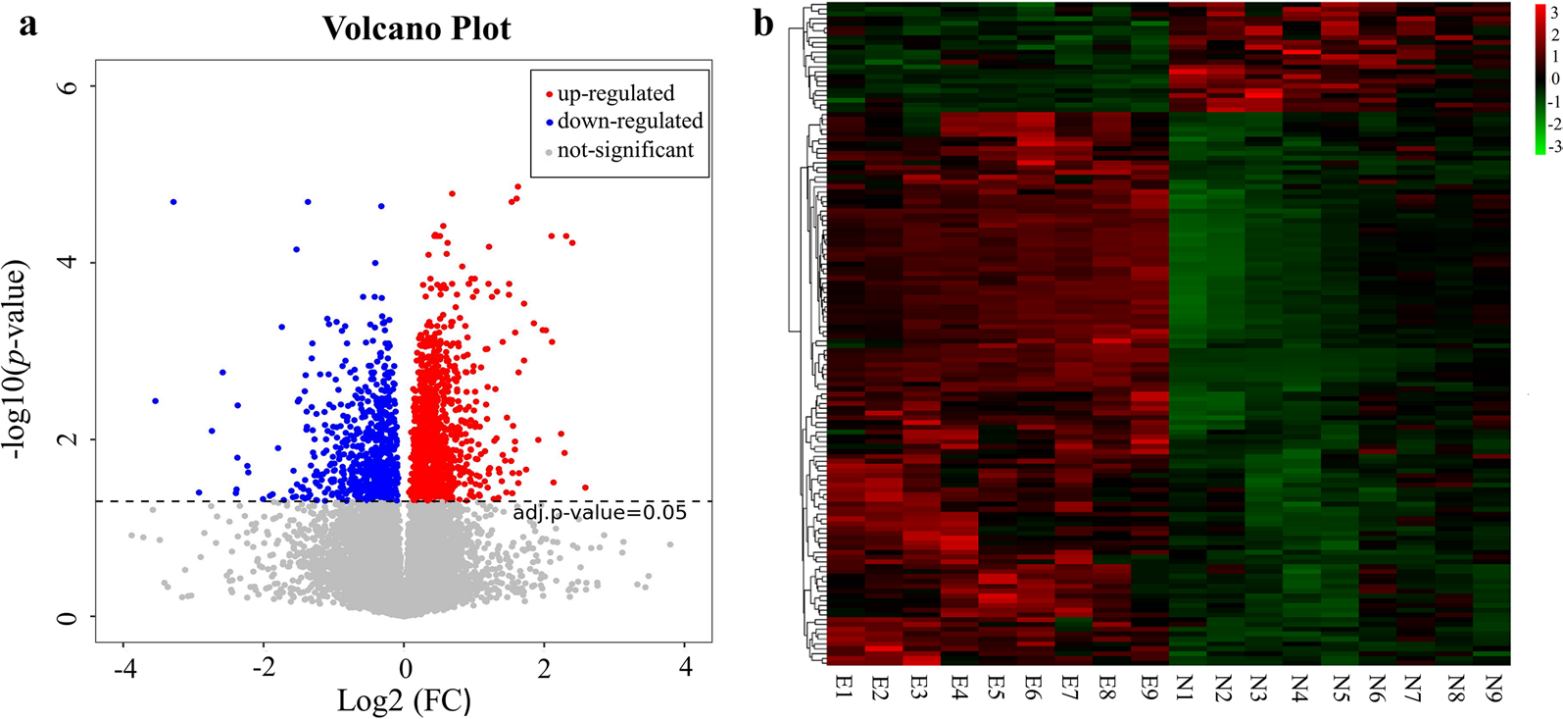
Expression levels of metabolites between the experimental (Case 1, E1–E9) and mock (Con 1, N1–N9) samples. **a** Volcano plot for all differential metabolites. Each dot represents one metabolite with detectable expression in both conditions, with the colored dots marking the threshold [false discovery rate (FDR) < 0.05] for defining a metabolite as differentially expressed. Red and blue points represent the significantly upregulated and significantly downregulated metabolites, respectively; gray points indicate non-significant differential metabolites. **b** Hierarchical cluster analysis of all differential metabolites (FDR < 0.05). Each sample is visualized in a single column and each metabolite is represented by a single row. Red coloration indicates significantly increased metabolite levels, while green coloration indicates low expression (see color scale on figure)

To explore the impact of *C. baileyi* infection on serum metabolism in chickens, the metabolic profiles in both groups of chickens were analyzed at 5 dpi. Using the criteria of VIP value > 1 in the OPLS-DA analysis and FDR (*q*-value) < 0.05 in the Benjamini–Hochberg method, we selected a total of 138 significantly differential metabolites (including 115 upregulated and 23 downregulated metabolites) (Fig. 2; Additional file 4: Table S3) following *C. baileyi* infection, corresponding to 1945 and 2290 for negative and positive ion modes, respectively. The top 20 significantly altered metabolites based on the VIP values are listed in Table 2. Further analysis showed that these metabolites could be grouped into 11 classified super classes (Additional file 4: Table S3), with lipids and lipid-like molecules being the most altered compounds in terms of metabolite numbers. Within this latter super class, 92 metabolites belonging to seven classes were identified, including glycerophospholipids (45), fatty acyls (20), sphingolipids (10), prenol lipids (7), steroids and steroid derivatives (4), sterol lipids (4) and glycerolipids (2).

**Table 2:** Top 20 serum metabolites following *C. baileyi* infection in chickens

Super class	Class	Metabolites	*m/z*	Ion mode	VIP score	FDR (*q*-value)	log_2_(FC)
Lipids and lipid-like molecules	Glycerophospholipids	PS(18:4(6Z,9Z,12Z,15Z)/18:4(6Z,9Z,12Z,15Z))	758.4397	Positive	7.1635	0.03205	0.6911
		PI(O-16:0/12:0)	758.5170	Positive	19.2165	0.002712	0.7590
		PI(18:1(11Z)/18:3(6Z,9Z,12Z))	857.5210	Negative	15.6296	4.986E−05	2.3144
		PI(16:2(9Z,12Z)/18:0)	833.5219	Negative	8.0607	4.986E−05	2.1052
		PI(16:0/20:4(5Z,8Z,11Z,14Z))	857.5225	Negative	7.2948	0.0005773	1.9798
		PI(16:0/18:2(9Z,12Z))	833.5214	Negative	12.2832	1.376E−05	1.6260
		PE-NMe2(16:0/18:2(9Z,12Z))	742.5401	Negative	7.3283	0.008647	0.3866
		PE(18:1(11Z)/16:0)	762.5106	Negative	8.4451	0.01716	0.3871
		PC(18:0/20:4(5Z,8Z,10E,14Z)(12OH[S]))	808.5876	Positive	11.4803	0.004282	0.3151
		PC(18:0/18:2(9Z,12Z))	830.5920	Negative	12.2456	0.005834	0.6030
		1-(8-[5]-ladderane-octanyl)-2-(8-[3] -ladderane-octanyl)-sn-glycerophosphoethanolamine	740.5361	Positive	6.8453	0.007720	0.4890
	Sphingolipids	Sphingosine 1-phosphate (d19:1-P)	808.5930	Positive	9.3068	0.0006339	0.4496
	Sterol lipids	3alpha,12alpha,15alpha-Trihydroxy-5beta-cholan-24-oic Acid	834.6075	Positive	8.1554	0.03314	0.3403
	Fatty acyls	Linoleamide	280.2623	Positive	11.4190	0.03024	0.2028
		Oleamide	563.5523	Positive	6.9537	0.004440	0.2195
		8E-Heneicosene	312.3620	Positive	7.4827	0.003657	0.2100
Unclassified	Unclassified	PC(14:0/22:1(13Z))	788.6176	Positive	19.1918	0.02364	1.3638
		GlcCer(t18:1(8Z)/18:0(2OH[S]))	782.5746	Positive	13.5588	0.01607	0.3220
		GlcCer(t18:1(8Z)/22:0(2OH[S]))	838.6411	Positive	11.4627	0.03009	0.4810
		Farnesyl acetone	263.2360	Positive	7.6243	0.009272	0.2245

*m/z* mass-to-charge ratio, *VIP* variable importance in projection,* FDR* false discovery rate,* log*_*2*_*(FC)* log_2_ fold change

The secondary super classes in metabolite numbers were organoheterocyclic compounds and organooxygen compounds, and 7 compounds were identified in both super classes. The former super class included azaspirodecane derivative (1), azoline (1), benzoxazine (1), diazanaphthalene (1), heteroaromatic compound (1), naphthofuran (1), and pyridines and derivative (1), and the latter super class only contained carbonyl compounds (7). Metabolites in other super classes were less than 7. Notably, 12 of differential metabolites could not be matched to any known super classes or classes (Additional file 4: Table S3).

### Metabolic pathway affected by *C. baileyi*

**Fig. 3:**
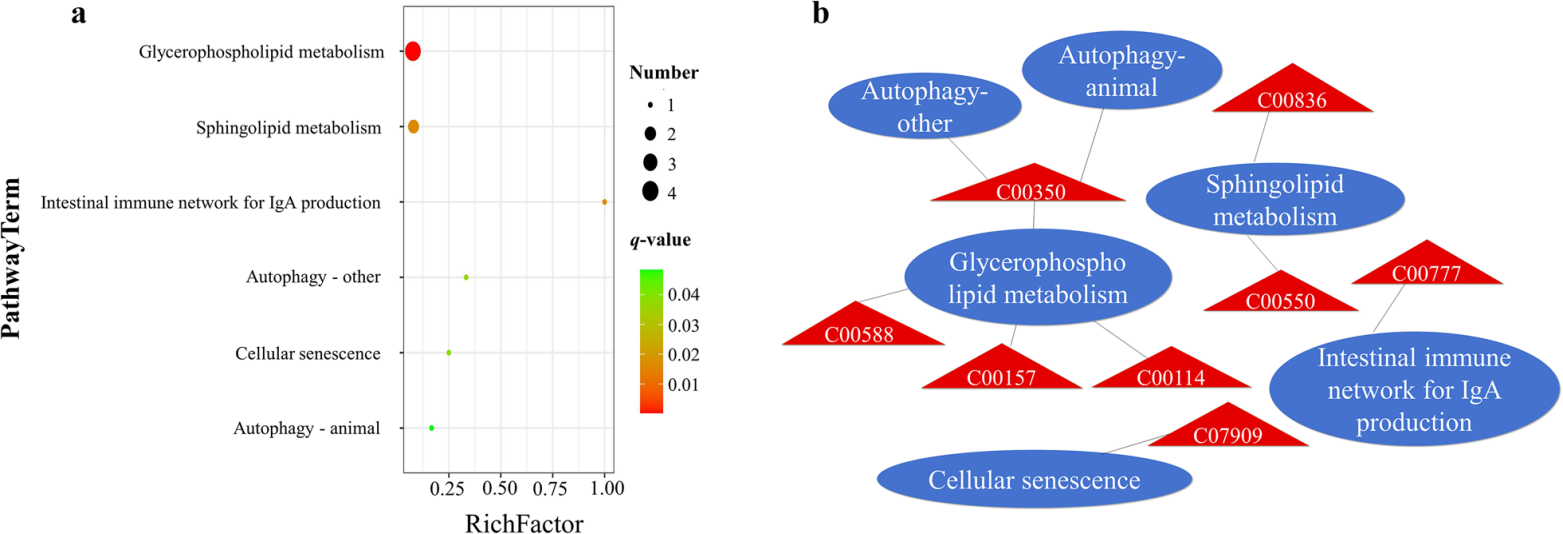
KEGG pathway enrichment analysis of differential serum metabolites following *C. baileyi* infection. **a** Significantly enrichments pathways with FDR (*q*-value) < 0.05. **b** Relationships between metabolic pathways and differential serum metabolites enriched. Each oval denotes one metabolic pathway. Triangles denote differentially abundant metabolites, with red representing upregulated metabolites

To understand potential functional significance of biological metabolisms during *C. baileyi* infection, all 138 differential metabolites identified in the present study were submitted into KEGG database for metabolic pathway enrichment analysis. Of 15 pathways detected, Six were found to be significantly different with FDR (*q*-value) < 0.05 by using the Benjamini–Hochberg method in infected chickens compared to mock birds (Fig. 3a**,** Additional file 5: Table S4). A total of 9 up-regulated metabolites were included into these significantly altered pathways (Fig. 3b**,** Additional file 6: Table S5).

Two most enriched pathways associated with energy and lipid metabolism were predicted following *C. baileyi* infection, namely glycerophospholipid metabolism (4 metabolites) and sphingolipid metabolism (2 metabolites). In addition, 3 metabolisms were also enriched into pathways involved into host immune response and defense against pathogenic agents, namely intestinal immune network for IgA production, autophagy, and cellular senescence.

### Identification of potential biomarkers

**Fig. 4:**
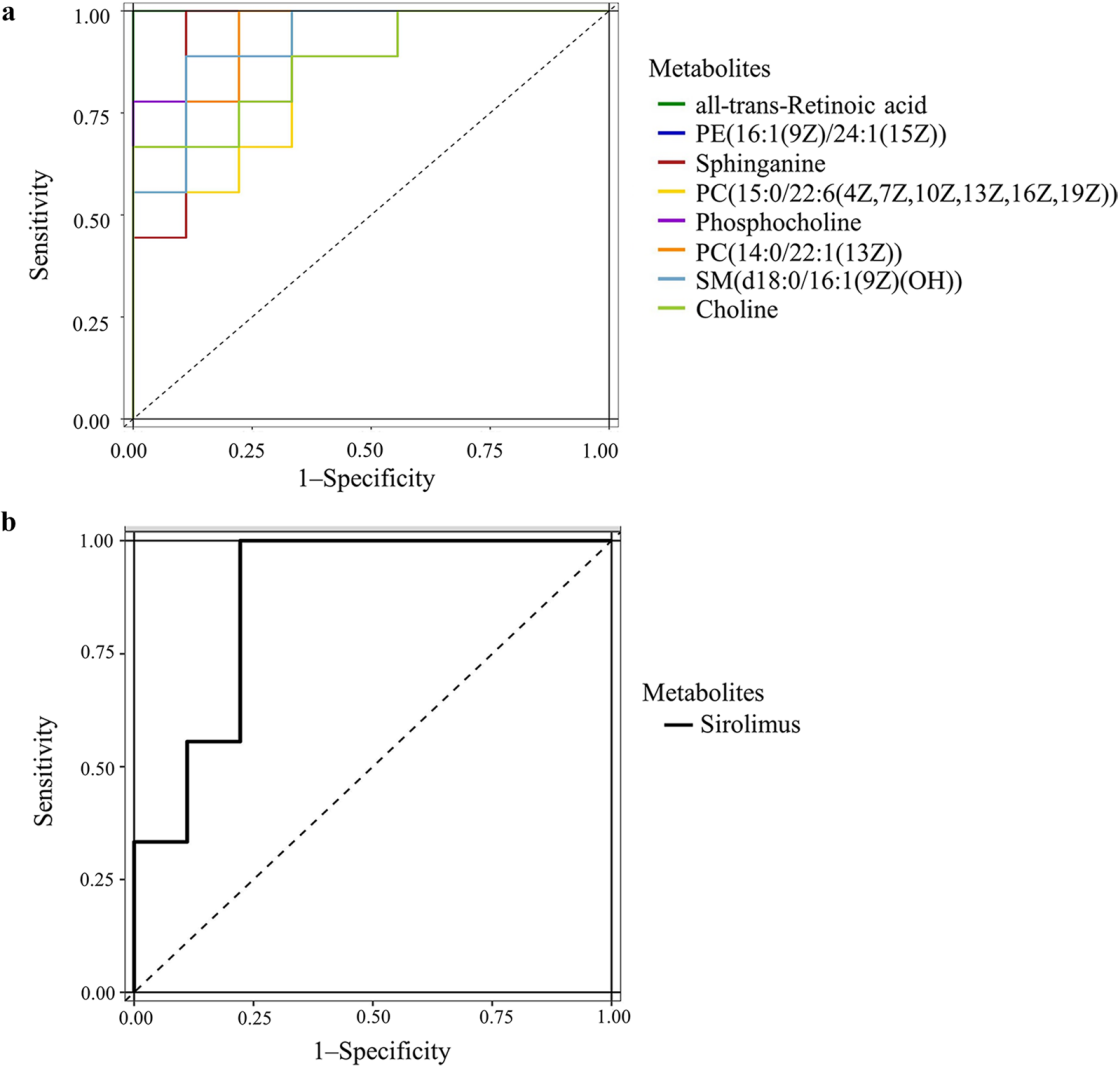
Identification of potential biomarkers response to *C. baileyi* infection. **a** Potential biomarker metabolites detected in ESI+ mode based on receiver operating characteristic curve analysis, **b** potential biomarker metabolites detected in ESI− mode based on ROC analysis.* ESI* Electrospray ionization

To identify the metabolic biomarkers related with *C. baileyi* early infection in chickens, all 9 metabolites enriched into significantly altered pathways, namely choline, sirolimus, all-trans-Retinoic acid, PC(14:0/22:1(13Z)), PC(15:0/22:6(4Z,7Z,10Z,13Z,16Z,19Z)), PE(16:1(9Z)/24:1(15Z)), phosphocholine, SM(d18:0/16:1(9Z)(OH)) and sphinganine, were selected for ROC analysis by using plotROC package [40]. We found all these metabolites with the AUC > 0.7 (Table 3), including 8 in ESI + mode (Fig. 4a) and 1 in ESI-mode (Fig. 4b).

**Table 3:** Potential serum biomarkers response to *C. baileyi* infection in chickens based on receiver operating characteristic curve analysis

Metabolites	KEGG ID	Ion mode	AUC	VIP	FDR (*q*-value)	log_2_(FC)	Pathways (FDR < 0.05)
All-trans-retinoic acid	C00777	Positive	1.000	1.542	0.002763	0.2638	Intestinal immune network for IgA production
PE(16:1(9Z)/24:1(15Z))	C00350	Positive	0.9012	1.184	0.01051	0.8962	Glycerophospholipid metabolism, Autophagy–other, Autophagy–animal
Sphinganine	C00836	Positive	0.9383	5.258	0.01546	0.2760	Sphingolipid metabolism
PC(15:0/22:6(4Z,7Z,10Z,13Z,16Z,19Z))	C00157	Positive	0.8642	1.155	0.02174	0.4605	Glycerophospholipid metabolism
Phosphocholine	C00588	Positive	0.9012	1.660	0.02200	0.3740	Glycerophospholipid metabolism
PC(14:0/22:1(13Z))	C00157	Positive	0.9383	19.19	0.02364	1.364	Glycerophospholipid metabolism
SM(d18:0/16:1(9Z)(OH))	C00550	Positive	0.9259	5.159	0.02786	0.3067	Sphingolipid metabolism
Choline	C00114	Positive	0.8765	1.257	0.04110	0.4412	Glycerophospholipid metabolism
Sirolimus	C07909	Negative	0.8765	2.448	0.04846	0.1805	Cellular senescence

*KEGG* *Kyoto Encyclopedia of Genes and Genomes*,* AUC* *area under the curve*

## Discussion

*Cryptosporidium baileyi* is a potential zoonotic *Cryptosporidium* species with specific parasitic sites (e.g. epithelial cells of the respiratory tract) that is distinct from gastrointestinal *C. parvum* and *C. hominis* and which can cause serious respiratory diseases in humans and birds [2, 11, 36]. It has been suggested that this protozoan parasite can be used as a model to characterize cryptosporidia due to its distinct morphological and biological features and large oocyst production in chickens [36, 41]. However, effective methods to prevent and control this parasite are currently lacking. Increasing evidence shows that metabolomics is an emerging “omics” technique that can provide a functional readout of cellular biochemistry for identifying significant metabolic pathways related to disease processes and potential biomarkers [42, 43], including metabolic diseases (e.g. diabetes, adiposity, metabolic syndrome), cancers (e.g. breast cancer, liver cancer) and microbial diseases (e.g. hepatitis B, tuberculosis) [15, 17, 19, 21, 22, 34, 44, 45]. In 2012, Ng et al. developed an untargeted metabolomics method using GC–MS to compare differences in metabolites present in fecal extractions from *Cryptosporidium*-positive and *Cryptosporidium*-negative patients [46], ultimately identifying 30 possible compounds that contributed most to the differences between two groups. Subsequently, metabolome changes following infections by zoonotic *C. parvum* and *C. hominis* were investigated in* in vivo* mouse models (pregnant female BALB/c mouse or neonatal Swiss mouse fecal sample extractions; neonatal ICR mice small intestinal luminal flush samples and cecal contents; protein-deficient diet C67BL/6 mouse urines) and in* in vitro* COLO-680 N cell culture using NMR [34], GC-TOF MS [47] or GC–MS [33], with the results showing that the host metabolic profile patterns response to *Cryptosporidium* infections were significantly affected by *Cryptosporidium* species (*C. parvum* or *C. hominis*) and isolates (*C. parvum* Iowa II or Weru), infection models, sample compartments (fece, urine, intestinal contents) and analytical platforms (NMR, GC–MS). In the present study, we used the UPLC-MS technique to explore the impact of *C. baileyi* infection on the serum metabolism of chickens at an early stage of infection. A total of 138 significantly differential serum metabolites were found following *C. baileyi* infection, most (115 differential metabolites) of which were upregulated. Of these, two upregulated organic nitrogen compounds, namely phosphocholine and choline, had previously been found to also be increased in the urine of C67BL/6 mice on a protein-deficient diet that were infected with *C. parvum* Iowa strain oocysts, at 7 dpi [47], and the up-regulation of choline was also detected in COLO-680 N cells infected with zoonotic *Cryptosporidium* spp. by using NMR [34]. Choline, an essential substrate in phosphatidylcholine and acetylcholine synthesis, is usually obtained from the diet or the disintegration of membrane phospholipids [48, 49]. Previous studies showed that choline played a significant role in signal transduction, neurotransmitter synthesis and regulation of lipid metabolism in human and animals (e.g. rodents, pigs and chickens) and that it was negatively correlated with weight gain in chickens [50–52]. Although few studies have reported the potential actions of host serum phosphocholine during pathogenic infection, the significance of microbial phosphocholine molecules has been demonstrated in host antibacterial immune response and diagnosis of bacterial infections [53–56].

Previous genomic and biochemical findings indicated that *Cryptosporidium* survival is highly dependent on host-derived biosynthetic pathways due to the unavailability of some key metabolic pathways and incapability to* de novo* synthesize nucleosides, fatty acids and amino acids [57, 58]. Significant alterations in metabolites of amino acid biosynthesis pathways have been revealed during *Cryptosporidium* infection [33, 34, 46, 47]. In the present study, we also detected two significantly differential amino acids, peptides or analogues in serum samples of chickens infected with *C. baileyi*, and N2-fructopyranosylarginine was upregulated. In addition, one fatty acid or conjugate and one carbohydrate or carbohydrate conjugate were found to be significantly decreased (Additional file 4: Table S3).

The KEGG pathway analysis identified three significantly altered metabolites enriched into four important immunity-associated signal pathways, including intestinal immune network for IgA production, autophagy and cellular senescence. Among these, the intestinal immune network for IgA production, which was the enriched metabolic pathway in this study, was also identified previously in the *C. baileyi* transcriptomic analysis of chicken tracheal tissues [36]. Specific serum IgA production was detected in patients infected with *C. parvum*, irrespective of human immunodeficiency virus/immune status [59], and specific IgA antibody response to the coproantigens of *C. parvum* was also found in serum samples in natural and experimentally infected calves [60, 61]. Serum IgA was also demonstrated in hens infected with *C. baileyi* [62]. In one study, although *Cryptosporidium*-induced autophagy-associated molecules were not investigated in host sera, autophagy occurred in intestinal epithelial cells following *C. parvum* infection [63]. Cellular senescence has been reported in enteroids isolated from neonatal mice and immunocompetent adults following *ex vivo C. parvum* infection, and senescent cells can communicate with immune cells to invoke an immune response against *C. parvum* by upregulation of the inflammatory genes *Mip-2*, *Nos2*, *Dkk1*, *Icam-1* and *IL-6* [64]. Notably, the significance of interplay between metabolic processes and immunity has been reported in several biological processes, including infectious diseases [65, 66]. For example, type I interferon signaling disrupted the hepatic urea cycle and altered systemic metabolism to suppress T-cell function in mice infected with chronic lymphocytic choriomeningitis virus (LCMV) [67]. A combination using genetics and metabolic profiling showed that nine of 12 compounds generated by the gut symbiont *Clostridium sporogenes* accumulated in host sera and that modulation of serum levels of these metabolites in gnotobiotic mice affected intestinal permeability and systemic immunity [68]. Of three significantly enriched metabolites in immunity-associated pathways during *C. baileyi* infection, PE(16:1(9Z)/24:1(15Z)), in addition to participating in glycerophospholipid metabolism, could initiate autophagy by covalently binding ATG8 during infections of foreign pathogens and homeostasis maintenance [69]. Rapamycin can inhibit the activity of mechanistic target of rapamycin (mTOR), decrease proliferation of T lymphocytes to reduce adipogenesis and enhance lipogenesis and induce tumor immune evasion [70, 71]. These findings indicate the potential roles of these differential metabolites and that the latter are involved in host immunity or immunopathogenesis during *C. baileyi* infection.

In recent years, metabolomics has been increasingly recognized as a novel promising tool for developing biomarkers for the early diagnosis of disease [72–74]. In our study, ROC analysis of significantly differential metabolites between two groups showed nine *C. baileyi*-responsive metabolites with AUC > 0.7, including choline, sirolimus, all-trans-retinoic acid, PC(14:0/22:1(13Z)), PC(15:0/22:6(4Z,7Z,10Z,13Z,16Z,19Z)), PE(16:1(9Z)/24:1(15Z)), phosphocholine, SM(d18:0/16:1(9Z)(OH)) and sphinganine. As an essential nutrient, choline can modulate immune response through one-carbon metabolism [75], and circulating choline together with its metabolites have been reported to be potential cardiometabolic biomarkers [76]. Sirolimus has been reported to be able to enrich several circulating pro-inflammatory factors, such as interleukin (IL)-12, IL-6 and IL-1β [77]. All-trans-retinoic acid has been found to play an important role in the differentiation of T cells and maintenance of homeostasis [78–83], and sphinganine has been identified to be required in programmed cell death, which is recognized as an effective strategy by which plants and animals can defend themselves against infections of pathogens [84]. Consequently, these differential compounds would be potential biomarkers for the early detection of *C. baileyi* in chickens and also could be used to reveal the suggested interactions between *C. baileyi* and its host (including chickens).

This study has a number of limitations. First, although several interesting metabolites were found to be responsive to infection by *C. baileyi*, the small number of chickens in the metabolomic analysis in our study was an unavoidable limitation. More experimental animals are needed to be included in future studies to further confirm our findings. Secondly, only one time point was selected in our study; as such, the dynamic metabolic process during the whole progression of infection can not be perfectly reflected, and limited metabolites of interest were obtained. Last but not the least, future studies should be conducted by integrated application of more available omics technologies (e.g. genomics, transcriptomics, proteomics) to analyze novel interesting findings in serum metabolic processes of chickens infected with *C. baileyi*. Solving these problems will advance our knowledge in the intricate interactions between *Cryptosporidium* and hosts.

## Conclusion

We have analyzed the serum metabolomics in chickens following *Cryptosporidium* infection at an early stage of infection for the first time and found differences in certain metabolites between infected birds and healthy ones. These differential compounds were mainly significantly enriched into energy and lipid metabolism and important immunity-associated signal pathways. Nine significantly metabolites were identified as compounds potentially responding to infection of *C. baileyi*, and these may be used to diagnose chicken cryptosporidiosis at an early stage. The findings in this study suggest that metabolomics profiling provides new insights that will deepen our understanding of the interplay between the host and *Cryptosporidium* and assist in the development of potential biomarkers for the early detection of *Cryptosporidium* infections in animals as well as humans.

## Supplementary Information

**Additional file 1: Table S1.** Ingredient composition and nutrient levels of the basal diets.

**Additional file 2: Figure S1.** Confirmation of *C. baileyi* infection in chickens. **a** Microscopic observation of oocysts in chicken feces of the experimental (left) and mock (right) group. Arrow indicates oocysts *of C. baileyi*. **b** Nested-PCR amplification results of *18S * rRNA gene of *Cryptosporidium*. Lanes: * M* DNA marker DL2000,* 1–9* fecal samples of E1–E9 in the experimental group, respectively,* 10–18* fecal samples of N1–N9 in the mock group, respectively,* 19* negative control. **c** Oocyst shedding of *C. baileyi* in chicken feces. The horizontal axis represents the day post infection (dpi) and the vertical axis shows the OPG

**Additional file 3: Table S2.** Metabolites in serum samples from mock and *C. baileyi*-infected chickens.

**Additional file 4: Table S3.** Differential metabolites between experimental and mock serum samples responsive to *C. bailey* infection identified by UPLC-MS in this study.

**Additional file 5: Table S4.** KEGG pathway analysis of all differential metabolites following *C. baileyi* infection in chickens.

**Additional file 6: Table S5.** KEGG ID to the differential metabolite name conversion table.

## Data Availability

Data supporting the conclusions of this article are included in the article.

## Abbreviations

AUC: Area under the ROC curve
dpi: Days post-infection
ESI: Electrospray ionization
FDR: False discovery rate
HMDB: Human Metabolome Database
GC–MS: Gas chromatography coupled to mass spectrometry
KEGG: Kyoto Encyclopedia of Genes and Genomes
LC–MS: Liquid chromatography coupled to mass spectrometry
log_2_FC: Log_2_ fold change
MSE: Mean square error
NMR: Nuclear magnetic resonance
NRC: National Research Council
OPLS-DA: Orthogonal partial least squares-discriminant analysis
OPG: Number of oocysts per gram
PCA: Principal component analysis
PLS-DA: Partial least squares-discriminant analysis
QC: Quality control
ROC: Receiver operating characteristic
RPT: Response permutation testing
RT: Retention time
UPLC-MS: Ultra-performance liquid chromatography-mass spectrometry
VIP: Variable Importance in Projection
