# Supplementary material for: Serum metabolomics in chickens infected with Cryptosporidium baileyi
Source: Parasit Vectors. 2021 Jun 26;14:336. doi: 10.1186/s13071-021-04834-y (PMC8235856; doi:10.1186/s13071-021-04834-y)
Supplement: Supplementary file 1 — Additional file 1: Table S1. Ingredient composition and nutrient levels of the basal diets. [file 13071_2021_4834_MOESM1_ESM.docx]

Table S1. Ingredient composition and nutrient levels of the basal diets

| Ingredient | Content (%) |
| --- | --- |
| Crude protein % ≥ | 21.0 |
| Crude ash % ≤ | 8.0 |
| Total phosphorus % | 0.50-0.75 |
| Moisture content % ≤ | 14.0 |
| Crude fiber % ≤ | 5.0 |
| Calcium % | 0.70-1.10 |
| Calcium chloride % | 0.30-0.80 |
| Methionine | 0.45 |

The test results of this product are in accordance with GB / T18823-2010

Note: Phytase Addition 1500 U/kg.

Raw material composition: corn, flour, soybean meal, cottonseed meal, corn gluten meal, stone powder, choline chloride, sodium chloride, calcium hydrogen phosphate, vitamin premix, trace-element premix, phytase etc.
