# Supplementary figures and images for: Serum metabolomics in chickens infected with Cryptosporidium baileyi
Source: Parasit Vectors. 2021 Jun 26;14:336. doi: 10.1186/s13071-021-04834-y (PMC8235856; doi:10.1186/s13071-021-04834-y)

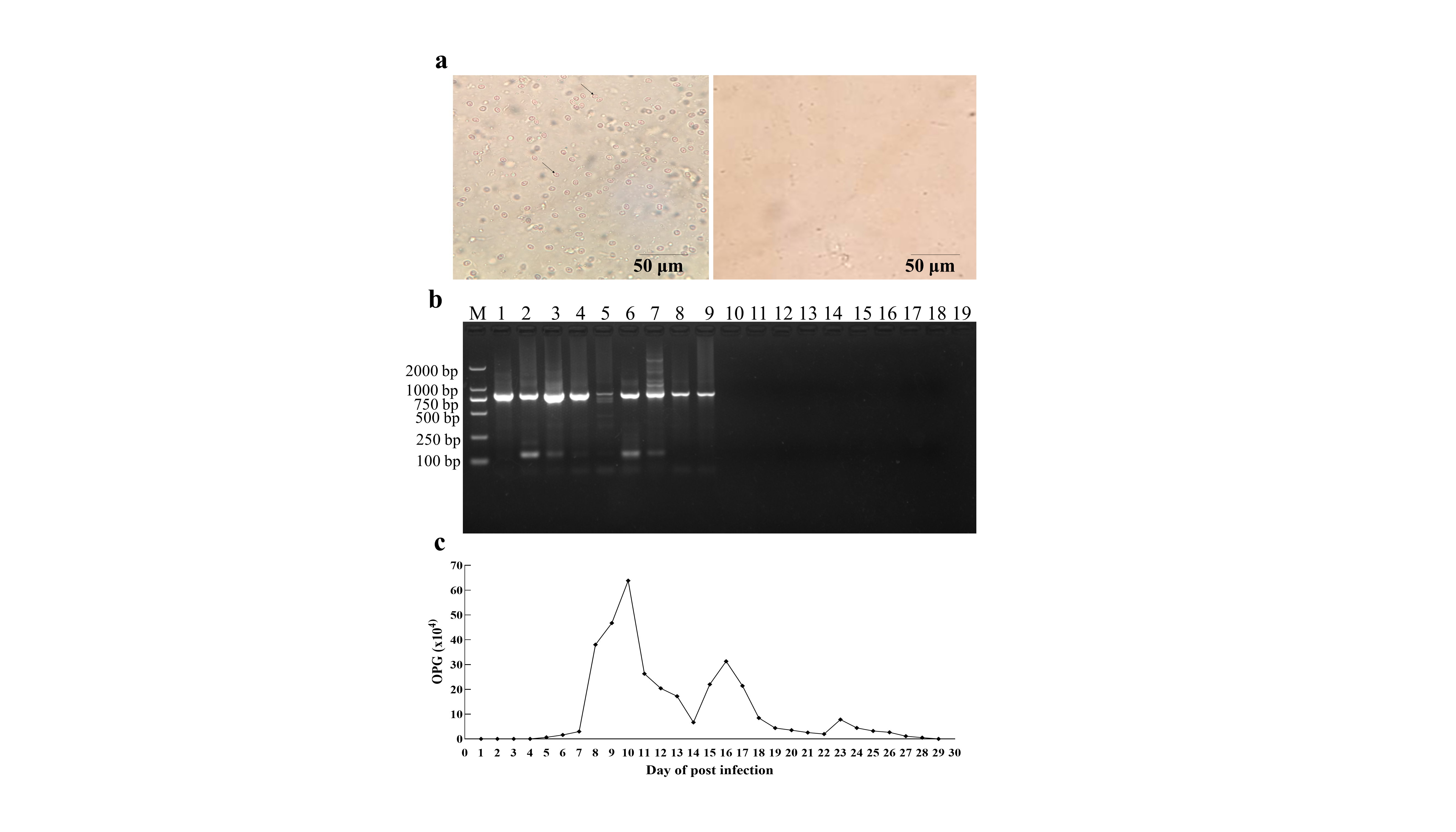

Supplement: Supplementary file 2 — Additional file 2: Figure S1. Confirmation of C. baileyi infection in chickens. a Microscopic observation of oocysts in chicken feces of the experimental (left) and mock (right) group. Arrow indicates oocysts of C. baileyi. b Nested-PCR amplification results of 18S rRNA gene of Cryptosporidium. Lanes: M DNA marker DL2000, 1–9 fecal samples of E1–E9 in the experimental group, respectively, 10–18 fecal samples of N1–N9 in the mock group, respectively, 19 negative control. c Oocyst shedding of C. baileyi in chicken feces. The horizontal axis represents the day post infection (dpi) and the vertical axis shows the OPG [file 13071_2021_4834_MOESM2_ESM.tif]
